# Surface, but Not Age, Impacts Lower Limb Joint Work during Walking and Stair Ascent

**DOI:** 10.3390/jfmk8040145

**Published:** 2023-10-13

**Authors:** Thomas A. Wenzel, Nicholas L. Hunt, Amy E. Holcomb, Clare K. Fitzpatrick, Tyler N. Brown

**Affiliations:** 1 Department of Kinesiology, Boise State University, Boise, ID 83725, USA; thomaswenzel@u.boisestate.edu (T.A.W.); nichunt@u.boisestate.edu (N.L.H.); 2 Department of Mechanical and Biomedical Engineering, Boise State University, Boise, ID 83725, USA; amyholcomb@u.boisestate.edu (A.E.H.);

**Keywords:** older adult, joint biomechanics, challenging surface, gait

## Abstract

Older adults often suffer an accidental fall when navigating challenging surfaces during common locomotor tasks, such as walking and ascending stairs. This study examined the effect of slick and uneven surfaces on lower limb joint work in older and younger adults while walking and ascending stairs. Fifteen young (18–25 years) and 12 older (>65 years) adults had stance phase positive limb and joint work quantified during walking and stair ascent tasks on a normal, slick, and uneven surface, which was then submitted to a two-way mixed model ANOVA for analysis. The stair ascent required greater limb, and hip, knee, and ankle work than walking (all *p* < 0.001), with participants producing greater hip and knee work during both the walk and stair ascent (both *p* < 0.001). Surface, but not age, impacted positive limb work. Participants increased limb (*p* < 0.001), hip (*p* = 0.010), and knee (*p* < 0.001) positive work when walking over the challenging surfaces, and increased hip (*p* = 0.015), knee (*p* < 0.001), and ankle (*p* = 0.010) work when ascending stairs with challenging surfaces. Traversing a challenging surface during both walking and stair ascent tasks required greater work production from the large proximal hip and knee musculature, which may increase the likelihood of an accidental fall in older adults.

## 1. Introduction

Every year more than 8 million older adults (65 years or older) suffer an accidental fall that requires medical attention [1]. Treating these falls annually costs more than $31.3 billion dollars yet fails to prevent further functional decline and long-term disability [2]. Age-related musculoskeletal issues, such as loss of skeletal muscle mass, may compromise older adults’ mobility and elevate their accidental fall risk. Specifically, the muscular weakness that occurs with advanced age manifests as unfavorable lower limb biomechanics changes, including diminished ankle joint torque and power, that may elevate accidental fall risk during common daily locomotor activities such as walking or stair negotiation [3,4]. Considering that 73% of accidental falls reportedly occur outdoors due to environmental factors [5], research that investigates whether navigating challenging surfaces, particularly slick and uneven conditions, exacerbates older adults’ unfavorable lower limb biomechanical adaptations is necessary to reduce the fall rate.

To compensate for lower limb muscular weakness, especially of the ankle plantar flexors, older adults rely upon the proximal leg musculature for mechanical power production (i.e., creating mechanical energy via concentric muscular contraction) during gait. Specifically, older adults exhibit up to a 40% decrease in maximal strength of their ankle joint musculature and subsequently walk with diminished ankle biomechanics, including up to 25% and 35% reductions in propulsive (i.e., plantarflexion) ankle joint torque and power production [6,7]. To maintain balance and overcome the diminished ankle contribution to forward propulsion, older adults compensate by reportedly producing greater power from the large proximal hip musculature [6,8,9]. When walking, for instance, older adults reportedly overcome the substantial reduction in ankle work by increasing hip power, producing 46% more work from their hip extensors than their younger counterparts [6,9,10]. Yet, the increased reliance on the proximal hip musculature with advanced age may not be sufficient to avoid an accidental fall, as older adults with a history of falling exhibit larger increases in power production from the hip musculature than non-fallers [11]. Considering that older adults reportedly have tight hip extensors, which may limit their range of motion and ability to adequately respond to an external perturbation, they may exhibit a lower limb biomechanical profile that limits their ability to avoid a fall when walking over challenging slick and uneven surfaces [12].

Ascending stairs requires greater mechanical work from the lower limb musculature. During a stair ascent, the knee is primarily responsible for lifting the center of mass up to the next step, and reportedly increases its work production by up to 56% to safely ascend steps [13,14]. Despite a similar knee kinematic profile when ascending stairs, older adults exhibit an approximate 35% reduction in peak knee extension moments, but a 24% increase in relative effort (the percentage of joint moment relative to maximum joint moment) compared to young adults [15]. When faced with ascending stairs, the increased requirement could cause issues if age-related weakness impairs their ability to generate the required amount of work. However, despite exhibiting compromised ankle plantar flexor strength, older adults can, indeed, increase power production during a similar uphill walking task. Specifically, older adults increased positive ankle work by 44%, and exhibited a similar increase in ankle work (0.09 J/kg vs. 0.08 J/kg) to young adults, when walking up a 9-degree incline [16]. Yet, it is relatively unknown whether older adults increase reliance on the proximal leg musculature to ascend stairs, or whether their tight hip extensors compromise their ability to adequately respond to stairs when surface conditions are challenging.

Navigating slick and/or uneven surfaces may challenge the neuromuscular system and further exacerbate unfavorable age-related changes in lower limb biomechanics. For instance, individuals exhibit adaptations in lower limb biomechanics to increase stability and mitigate fall risk, including slower, shorter strides with greater lower limb flexion and muscle co-contraction, when walking over a challenging surface [17,18]. Older adults reportedly exhibit larger, more pronounced biomechanical changes when walking over an uneven surface than their younger counterparts [19]. Specifically, older adults purportedly use slower, more variable strides with greater knee and ankle flexion posture and co-contraction of the associated musculature on challenging surfaces [18,20]. Yet, the reported biomechanical changes due to challenging surfaces may be associated with strength of the lower limb musculature [21]. Considering older adults have a documented decrement in lower strength compared to their younger counterparts, they may exhibit a greater reliance proximal leg musculature to walk and ascend stairs with a challenging slick or uneven surface. Because it is currently unknown whether navigating uneven and slick surfaces leads to proximal redistribution of lower limb work, we tested the following hypotheses. Specifically, we hypothesize that older adults will produce less limb and joint work than young adults, with greater proximal joint contribution when walking and less knee contribution when ascending stairs; while all participants (young and older) will increase the contribution of proximal joint work to walk on or ascend stairs fitted with a challenging surface. Therefore, the study determined differences in lower limb work for young and older adults during an over-ground walk and stair ascent, and whether they differ when traversing challenging surfaces.

## 2. Materials and Methods

Fifteen young (between 18 and 25 years of age) and 12 older (over 65 years of age) adults participated (Table 1). To meet the study inclusion criteria, young adults had to self-report no history of musculoskeletal injury or disease, while older adults had to self-report one accidental fall in the past 12 months. Potential participants that self-reported (1) past lower extremity back surgery or injury, (2) recent lower extremity or back pain or injury, and/or (3) known neurological dysfunction were excluded. Every attempt was made to match young and older adults by sex and body mass index. Prior to testing, the local Institutional Review Board provided research approval and each participant provided written informed consent.

For testing, each participant had three-dimensional (3D) lower limb (hip, knee, and ankle) biomechanical data recorded during a walk and stair ascent task. During each task, one force platform (2400 Hz, AMTI OR6 Series, Advanced Mechanical Technology Inc., Watertown, MA, USA) recorded ground reaction force (GRF) data, while ten high-speed optical cameras (240 Hz, Vantage, Vicon Motion Systems, Ltd., Oxford, UK) recorded lower limb motion data. Throughout testing, participants wore spandex shirt and shorts, broken-in tennis shoes, and a safety harness attached to an overhead gantry via a cable to prevent an accidental fall.

To quantify dominant lower limb biomechanical data, the 3D coordinates of 50 retro-reflective and four virtual markers were recorded during each walk and stair ascent trial. Each retro-reflective marker was placed and secured at specific anatomical landmarks, while virtual markers were digitized in the global coordinate system over specific anatomical landmarks (Davis Digitizing Pointer, C-Motion Inc., Rockville, MD, USA). With all markers visible, participants stood in anatomical position to capture a static recording used to create a kinematic model in Visual 3D (v6, C-Motion, Inc., Germantown, MD, USA). The kinematic model consisted of trunk, pelvis, and bilateral thigh, shank, and foot segments, with 27 degrees of freedom, according to previous work [20].

For the walk task, participants walked at a self-selected speed through the motion capture space (about 10 m) and contacted the force platform with their dominant limb. For the stair ascent task, participants walked at a self-selected speed to ascend two stairs (18.5 cm rise) fixated atop the force platform. Specifically, the stair ascent task required walking through the motion capture space, and then placing their dominant foot on the first step, before ascending to the second step. Stair height was set according to the requirements of the 2021 International Residential Code [22]. A participant’s self-selected walk speed was established prior to testing by having each participant walk at a comfortable speed through the motion capture space five times. Two sets of infrared timing gates (TracTronix TF100, TracTronix Wireless Timing Systems, Lenexa, KS, USA), placed 1.8 m apart, recorded the walk speed of each trial, and the self-selected speed was calculated as the average of the five trials. Prior to testing, foot dominance was also determined by asking the participant which foot they would use to kick a ball [23].

**Figure A1:**
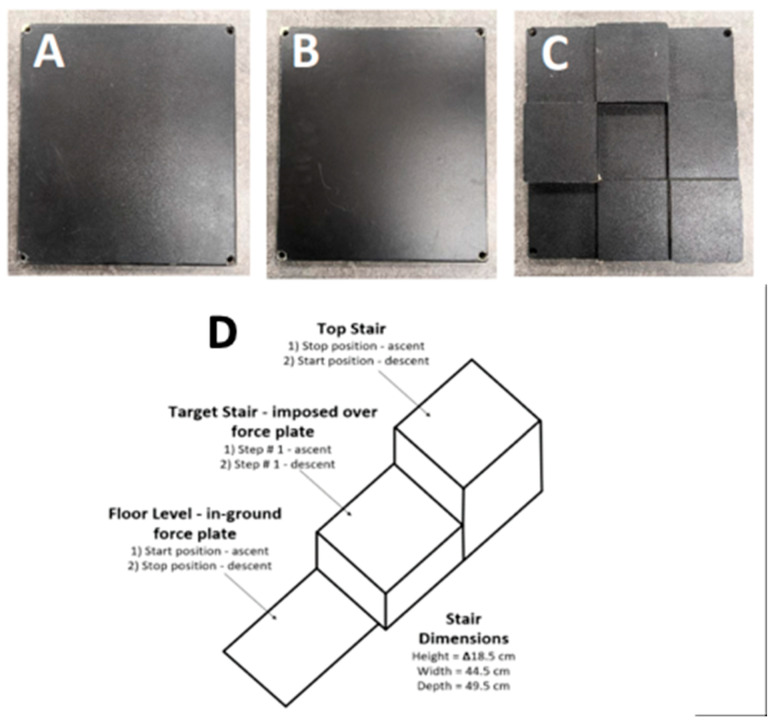
Depicts normal (**A**), slick (**B**), and uneven (**C**) surfaces fixed on the target and top stair of the staircase (**D**). This figure was modified from Hunt, Nicholas Lynn, “Effects of Aging on Patellofemoral Joint Stress During Stair Negotiation on Challenging Surfaces” (2022). Boise State University Theses [44].

Each task was performed on three surfaces (normal, slick, and uneven) secured atop the force platform or stair surface (Figure A1). The normal surface consisted of a flat and painted wood panel. The slick surface task consisted of a wood panel covered with a smooth, plastic material that, when combined with the slick booties each participant was required to wear, produced a coefficient of friction (0.19) between the shoes and surface that was comparable to ice [24]. The uneven surface consisted of nine wooden blocks of differing heights attached to a wood panel. Participants completed three successful trials over each surface for each task. For a trial to be considered successful, the participant must have completed each task without slipping or tripping, only contacted the force platform or stair surface with their dominant limb and walked within ±5% of their self-selected speed. Prior to testing, the task and surface test order were randomly assigned to each participant to avoid bias and confounding data.

For each walk and stair ascent trial, synchronous GRF and marker trajectory data were low-pass filtered with a fourth-order Butterworth filter (12 Hz). Using Visual 3D, the filtered marker trajectories were processed to calculate lower limb joint rotations, and the filtered kinematic and GRF data were processed to obtain lower limb joint moments. Lower limb joint power was calculated by multiplying each lower limb joint moment by its respective joint angular velocity. Custom MATLAB (R2021b, Mathworks, Natick, MA, USA) code was used to calculate positive limb and joint mechanical work. To determine positive work, each joint power was time-integrated across the stance phase and sum of positive mechanical work was calculated. Total positive limb work was defined as the sum of positive mechanical work completed at each joint during stance. Joint contribution to positive limb work was defined as percent contribution to total limb work (joint work divided by limb work and multiplied by 100). Stance phase was defined as heel-strike to toe-off, or when GRF first exceeded and fell below 10 N. All biomechanical data were normalized from 0 to 100% of stance phase and resampled in 1% increments (*n* = 101). To make comparisons between participants, joint power and work were normalized to participant body mass (W/kg, J/kg).

Joint and limb positive work and relative joint work were submitted to statistical analysis. Each dependent variable was averaged across the three successful trials for each task. Each dependent variable was submitted to a two-way mixed model ANOVA to test the main effects of and interaction between age (young vs. older adults) and surface (normal, slick, and uneven) for the walk and stair ascent task, respectively. Significant interactions were submitted to simple effects analysis, and a Boneferroni correction was used for pairwise comparisons [25]. In addition, a paired t-test determined whether each dependent variable differed between the walk and stair ascent tasks. Statistical analysis was conducted using SPSS (v28, IBM, Armonk, NY, USA), with alpha level established a priori as 0.05.

## 3. Results

**Figure 1:**
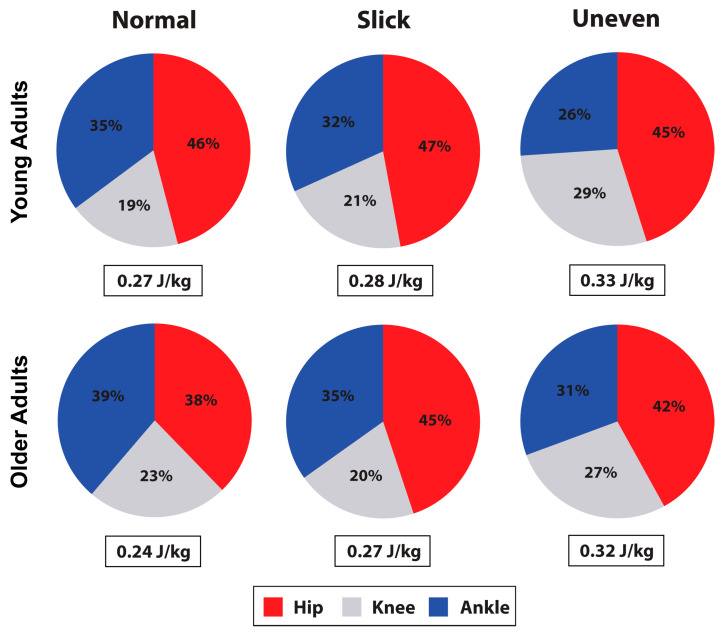
Limb positive work (J/kg) observed in young and older adults during the walk, and percent contribution to total limb work by the hip, knee, and ankle.

**Figure 2:**
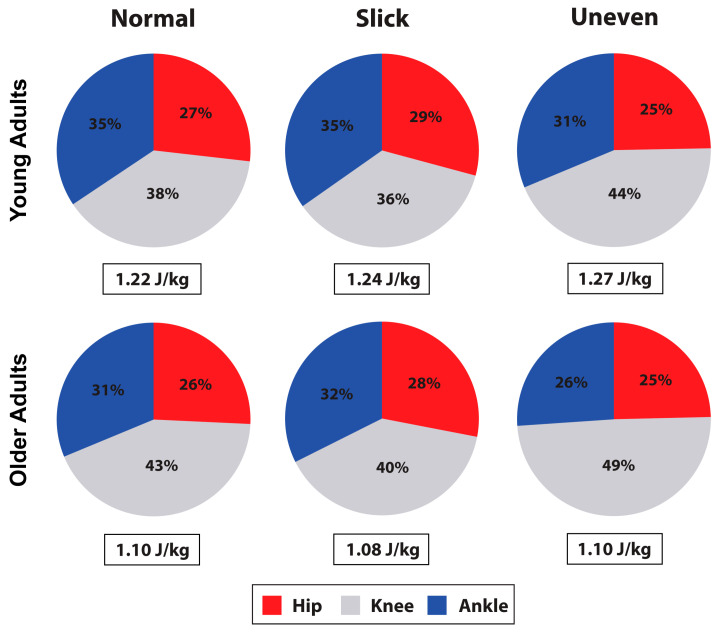
Limb positive work (J/kg) observed in young and older adults during the stair ascent, and percent contribution to total limb work by the hip, knee, and ankle.

Participants produced more positive limb, hip, knee, and ankle work during the stair ascent compared to walking (all: *p* < 0.001) (Table 2 and Table 3). During the walk, participants produced a greater percent contribution to total average positive power at the hip (*p* < 0.001), whereas, during the stair ascent, they produced a greater percent contribution at the knee (*p* < 0.001) (Figure 1 and Figure 2). 

**Figure 3:**
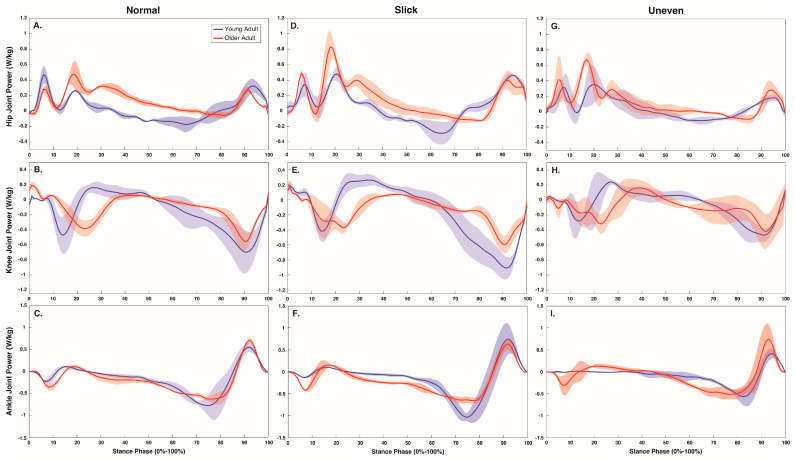
Stance phase (0–100%) hip (**A**,**D**,**G**), knee (**B**,**E**,**H**), and ankle (**C**,**F**,**I**) joint power (W/kg) over each surface (normal, slick and uneven) during the walk.

During the walk, surface impacted limb (*p* < 0.001), hip (*p* = 0.010), and knee (*p* < 0.001) positive work production (Table 2, and Figure 1 and Figure 3). On the uneven surface, participants produced more limb (*p* < 0.001 and *p* = 0.010) and knee (*p* = 0.001 and *p* = 0.001) work compared to normal and slick surfaces, and more hip work (*p* = 0.044) compared to normal surface. Hip work production was also greater on slick compared to normal surface (*p* = 0.006). Percent knee (*p* = 0.041) and ankle (*p* < 0.001) contribution to total average power was also impacted by the surface (Figure 1). Specifically, the knee produced a greater percent contribution on the uneven surface than on the slick surface (*p* = 0.031), while ankle produced a greater contribution on the normal compared to the uneven (*p* < 0.001) and slick (*p* = 0.047) surfaces, with a greater contribution on the slick surface than on the uneven surface (*p* = 0.027). Age did not impact lower limb work production (*p* > 0.05) during the walk (Table 2).

**Figure 4:**
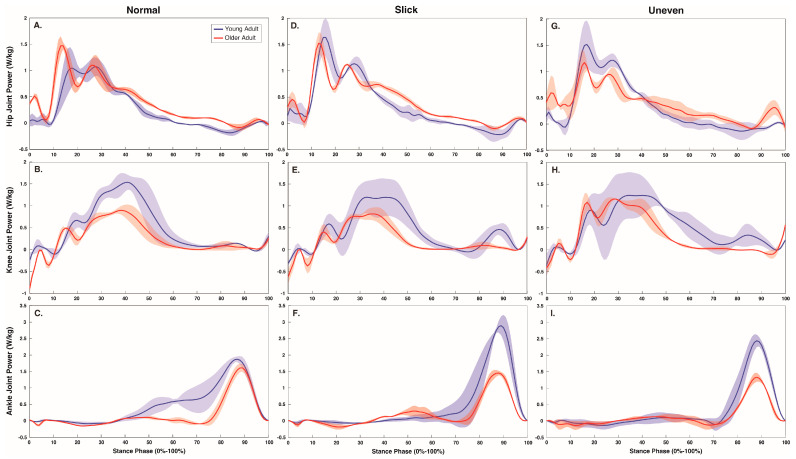
Stance phase (0–100%) hip (**A**,**D**,**G**), knee (**B**,**E**,**H**), and ankle (**C**,**F**,**I**) joint power (W/kg) over each surface (normal, slick, and uneven) during the stair ascent.

During the stair ascent, surface impacted hip (*p* = 0.015), knee (*p* < 0.001), and ankle (*p* = 0.010) positive work production (Table 3, and Figure 2 and Figure 4). Both hip and ankle produced greater work on the slick surface than on the normal surface (*p* = 0.007 and *p* = 0.046), while the knee produced greater work on the uneven compared to the normal (*p* = 0.005) and slick (*p* < 0.001) surfaces, with greater work on the normal surface than on the slick surface (*p* = 0.04). The percentage of hip, knee, and ankle contribution to total average power was also impacted by surface (all *p* < 0.001) (Figure 2). The percentage hip contribution was higher on the slick compared to the normal (*p* = 0.029) and uneven (*p* = 0.005) surfaces. The percentage knee contribution was higher on the uneven compared to the normal (*p* = 0.003) and slick (*p* < 0.001) surfaces and was higher on the normal than on the slick (*p* = 0.003) surface. At the ankle, percentage contribution was greater on normal and slick surfaces compared to uneven surface (both *p* < 0.001). Age did not impact lower limb work production (*p* > 0.05) during the stair ascent (Table 3).

## 4. Discussion

This study examined lower limb work as young and older adults walked and ascended stairs over challenging slick and uneven surfaces. Contrary to our hypothesis, older adults did not alter their lower limb work production compared to young adults. Yet, in agreement with our hypothesis, the challenging surfaces led to greater lower limb work production during both walking and stair ascent, particularly from proximal hip and knee joints.

Ascending stairs, regardless of age, required greater lower limb work than walking. Participants currently exhibited between 84% and 163% greater work from the hip, knee, and ankle to ascend the stairs compared to over-ground walking. In agreement with previous literature, participants increased the positive work from knee musculature to safely lift the center of mass during the stair ascent, with the joint producing 48% more work than during the over-ground walking [14]. The increased demand on knee musculature during stair ascent could be problematic for older adults as it may accelerate muscular fatigue, elevating the likelihood of an accidental fall from failing to properly raise the center of mass [3]. Despite requiring less positive mechanical work than stair ascent, walking may also elevate fall risk, particularly for older adults, as it required 37% more work from hip musculature to maintain task forward propulsion. Hip extension is essential for forward gait propulsion, as it assists the ankle, particularly during terminal stance, with safely propelling the center of mass forward into the next base of support [26,27]. Considering that older adults, particularly those with a history of an accidental fall, walk with approximately 59% less hip extension, their reduced hip range of motion may constrain the associated musculature’s contribution to forward propulsion and contribute to the purported decrease in mobility and elevated fall rates exhibited by older adults during walking [12].

Contrary to our hypothesis and previous literature, older adults did not alter lower limb work magnitude and distribution compared to their younger counterparts during either task [6,9]. Older adults are reported to walk with 46% more hip work and ascend stairs with 24% greater knee relative effort [9,15]. Yet, the older adults currently exhibited a non-significant decrease of approximately 31% and 8% in hip and knee work to walk and ascend stairs compared to the young adults. While the reason for the current discrepancy is not immediately evident, it may stem from the activity level of the older adult cohort. Although the current older adults had to be over 65 years of age and self-report one accidental fall in the previous 12 months to be included, potential participants were not excluded due to physical activity, despite the fact a sizeable number of participants came from an older-adult community that aims to keep individuals active. Considering walking speed typically decreases with age but may increase with physical activity [28], the current older adults, who did not exhibit a significant difference in self-selected walk speed (Table 1, 1.05 ms vs. 1.07 ms) compared to their younger counterparts, may be more active than the typical older adult cohort. Physical activity helps older adults maintain lower limb strength, improve joint proprioception and musculature activation, and decrease muscle fatigability [29,30,31]. As such, the current, active older adult cohort may better utilize their lower limb musculature than sedentary older adults in general. Specifically, they may better utilize their ankle musculature to produce work for walk forward propulsion and knee musculature to lift the center of mass during a stair ascent. Older adults, however, are reportedly able to exhibit similar increases in ankle work to young adults (0.09 J/kg vs. 0.08 J/kg) during a similar uphill walk task [16]. Further research is needed to determine the neuromuscular profile necessary for older adults to adequately respond to challenging external perturbations and mitigating age-related reductions in mobility and to reduce the likelihood of suffering an accidental fall during common locomotor activities.

Walking over a challenging, particularly an uneven, surface required greater lower limb work. On the uneven surface, participants increased limb work production by up to 20%. The greater limb work required to walk over the challenging, uneven surface may stem from an increased contribution from the large, proximal hip and knee musculature, or concurrent increases of 16% and 55% in, respectively, hip and knee work. Considering older adults primarily rely on the large proximal hip and knee musculature to walk [8], which is more likely to suffer age-related decrements, such as loss of mass and strength, or disease [32], navigating challenging surfaces may be a greater hindrance to older adult mobility. In particular, the increased mechanical work of the large hip and knee musculature required to safely walk across a challenging uneven surface may accelerate older adult muscular fatigue, constrain their forward propulsion, and increase their likelihood of suffering an adverse event, such as a slip or fall. Yet, in agreement with previous literature, the increased hip and knee work was offset by a concurrent reduction in ankle kinetics [21,33]. Specifically, when walking over challenging surfaces, the current participants exhibited an approximate 3% to 9% reduction in work contribution from the ankle musculature. Typically, the ankle is a primary producer of forward propulsion and reductions in ankle power generation may impair an individual’s mobility [8,34]. However, considering that navigating the challenging slick and uneven surfaces would introduce an external perturbation that compromises an individual’s stability, further research is needed to determine whether the reductions from the ankle musculature are a compensatory strategy to decrease fall risk.

Ascending stairs fitted with a challenging surface required greater hip, knee, and ankle work. Interestingly, during the stair ascent, the slick surface led to 14% and 3% greater hip and ankle work, while the uneven surface required 16% more knee work. The instability created by the slick surface reportedly requires a greater contribution from the distal ankle musculature to stabilize the foot and prevent an accidental slip [35,36]. As such, when ascending a slick stair, the small, significant increase in work from the biarticular ankle musculature may act to stabilize the ankle and contribute to maintaining a joint posture to minimize slip risk, rather than generating knee work to lift the center of mass; however, this would require the concurrent 14% increase in hip work to ascend to the next stair. The challenge posed by the uneven stairs, however, may destabilize the ankle and prevent the individual from adopting the stable, flat foot [17,21]. Therefore, this stability may require a further increase in work by the knee musculature to lift the center of mass, as evidenced by the current 16% increase in knee work observed to ascend the uneven steps. Yet, individuals who are prone to weakness of the knee musculature, such as older adults, may have difficulty safely completing the tasks and be more likely to suffer an accidental fall when ascending uneven steps [37]. 

One possible study limitation is the older adult participants. Although older adults are typically reported to walk more slowly than younger adults [38], the current self-selected walk speed did not significantly differ between young and older cohorts. Considering walking speed is purported to impact lower limb power [6,8], the self-selected walk speed of the current older adults may obfuscate “true” lower limb work and power differences between young and older adults. However, the self-selected speed is representative of daily life and the current analysis provides valuable insight into the importance of physical activity in maintaining mobility as individuals age. Another possible limitation is the chosen surfaces. Despite trying to accurately mimic uneven and slick surfaces encountered in everyday locomotor activities, it is possible that neither surface elicited the instability and/or randomness found in real-world terrain. Finally, the use of external skin markers for quantifying rigid body kinematics may be a limitation. Although the underlying rationale for using rigid body kinematics is to record trajectories of body motions with skin-based markers [39], there is the potential for excessive skin movement to result in erroneous estimation of rigid body motion [40,41]. However, we took several steps to minimize this problem. First, we negated the potential for significant intertester error by having a single investigator (AEH) place markers on all participants [42]. Second, to calculate rigid body kinematics, we utilized Visual 3D software, which relies upon a least squares global optimization technique that better exploits redundancy in the marker set and produces results less sensitive to errors in marker trajectories [43]. While we acknowledge excessive skin movement is largely unavoidable, we are confident that any subsequent errors have not confounded the current study outcomes, as our rigid body kinematics were consistent across conditions.

## 5. Conclusions

In conclusion, ascending stairs required more lower limb work than walking and greater contribution from the knee musculature, which may increase fall risk—particularly for older adults. However, age did not impact lower limb work production during either the walk or stair ascent tasks. Traversing a challenging surface during both walk and stair ascent tasks leads to an increased contribution from the large proximal hip and knee musculature, which may increase the likelihood of suffering an accidental fall—particularly for individuals prone to muscular weakness, like older adults.

## Figures and Tables

**Table 1:** Mean (SD) subject demographics for each cohort (young and older adults).

	N	Age (yrs)	Height (m)	Weight (kg)	Walk Speed (m/s)
Young	15	21.67 (2.12)	1.75 (0.10)	71.39 (15.54)	1.07 (0.74)
Older	12	70.08 (2.95)	1.72 (0.12)	75.05 (16.62)	1.05 (0.16)

**Table 2:** Mean (SD) for positive lower limb and joint work (J/kg) during the walk task for young and older adults on each surface.

	Normal		Uneven		Slick	
	Young	Older	Young	Older	Young	Older
Total Limb Work (J/kg) *	0.27 (0.10)	0.24 (0.07)	0.33 (0.13)	0.32 (0.10)	0.28 (0.11)	0.27 (0.09)
Hip Positive Work (J/kg) *	0.12 (0.05)	0.09 (0.04)	0.15 (0.07)	0.13 (0.06)	0.13 (0.06)	0.12 (0.04)
Knee Positive Work (J/kg) *	0.04 (0.03)	0.06 (0.03)	0.09 (0.05)	0.10 (0.06)	0.06 (0.03)	0.06 (0.03)
Ankle Positive Work (J/kg)	0.10 (0.04)	0.09 (0.04)	0.10 (0.07)	0.09 (0.05)	0.09 (0.04)	0.09 (0.05)

* Denotes a significant (*p* < 0.05) main effect of surface.

**Table 3:** Mean (SD) for positive lower limb and joint work (J/kg) during the stair ascent task for young and older adults on each surface.

	Normal		Uneven		Slick	
	Young	Older	Young	Older	Young	Older
Total Limb Work (J/kg)	1.22 (0.45)	1.10 (0.35)	1.27 (0.44)	1.10 (0.32)	1.24 (0.47)	1.08 (0.29)
Hip Positive Work (J/kg) *	0.30 (0.15)	0.30 (0.11)	0.30 (0.16)	0.27 (0.11)	0.35 (0.17)	0.31 (0.11)
Knee Positive Work (J/kg) *	0.50 (0.20)	0.46 (0.27)	0.58 (0.21)	0.52 (0.23)	0.46 (0.20)	0.42 (0.19)
Ankle Positive Work (J/kg)	0.42 (0.21)	0.35 (0.11)	0.38 (0.20)	0.31 (0.12)	0.43 (0.20)	0.35 (0.11)

* Denotes a significant (*p* < 0.05) main effect of surface.

## Data Availability

The data presented in this study are openly available in the following data repository [doi: 10.18122/cobr_data/4/boisestate].
